# The Paris pledges and the energy-water-land nexus in Latin America: Exploring implications of greenhouse gas emission reductions

**DOI:** 10.1371/journal.pone.0215013

**Published:** 2019-04-16

**Authors:** Silvia R. Santos Da Silva, Fernando Miralles-Wilhelm, Raul Muñoz-Castillo, Leon E. Clarke, Caleb J. Braun, Alison Delgado, James A. Edmonds, Mohamad Hejazi, Jill Horing, Russell Horowitz, Page Kyle, Robert Link, Pralit Patel, Sean Turner, Haewon C. McJeon

**Affiliations:** 1 Department of Atmospheric and Oceanic Science, University of Maryland, College Park, Maryland, United States of America; 2 Joint Global Change Research Institute, Pacific Northwest National Laboratory, College Park, Maryland, United States of America; 3 Earth System Science Interdisciplinary Center, University of Maryland, College Park, Maryland, United States of America; 4 Inter-American Development Bank, Washington DC, United States of America; University of Georgia, UNITED STATES

## Abstract

In the 2015 Paris Agreement, nations worldwide pledged emissions reductions (Nationally Determined Contributions—NDCs) to avert the threat of climate change, and agreed to periodically review these pledges to strengthen their level of ambition. Previous studies have analyzed NDCs largely in terms of their implied contribution to limit global warming, their implications on the energy sector or on mitigation costs. Nevertheless, a gap in the literature exists regarding the understanding of implications of the NDCs on countries’ Energy-Water-Land nexus resource systems. The present paper explores this angle within the regional context of Latin America by employing the Global Change Assessment Model, a state-of-the-art integrated assessment model capable of representing key system-wide interactions among nexus sectors and mitigation policies. By focusing on Brazil, Mexico, Argentina and Colombia, we stress potential implications on national-level water demands depending on countries’ strategies to enforce energy-related emissions reductions and their interplays with the land sector. Despite the differential implications of the Paris pledges on each country, increased water demands for crop and biomass irrigation and for electricity generation stand out as potential trade-offs that may emerge under the NDC policy. Hence, this study underscores the need of considering a nexus resource planning framework (known as “Nexus Approach”) in the forthcoming NDCs updating cycles as a mean to contribute toward sustainable development.

## Introduction

The “Nexus Approach” was defined by [1] as a conceptual paradigm to tackle the inherent linkages among the energy, water, food and land sectors. This novel concept has helped to identify critical barriers to a more efficient governance across sectors in light of the escalating human demands and climate change.

Particularly in Latin America, interest in nexus issues has also been motivated by some key domestic characteristics: great dependence on the water supply (abundant in total, albeit with large spatial and temporal heterogeneities) that can transfer climate change impacts to several sectors, importance of agriculture to local economies (whose expansion has been historically based on excessive exploitation of natural resources), and lower adaptive capacity to climate change compared to developed economies.

Given the multitude of nexus interconnections occurring in a wide range of temporal and spatial scales, a growing body of literature has recognized that governance of nexus resources should evolve from the current view centered on only one or two of these sectors toward an integrated nexus approach of planning and management. Such paradigm aims at: ensuring economic and resource efficiency, avoiding unintended competition for nexus resources, and capturing vulnerabilities across the three systems [2–4].

While general awareness of nexus issues has increased throughout this decade, a major societal concern has been how to overcome the challenge of significantly curbing anthropogenic greenhouse gas (GHG) emissions by the end of the 21^st^ century. In this sense, the 2015 Paris Climate Agreement brought the United Nations Framework Convention on Climate Change (UNFCCC) member states to put forward actions to keep global warming well below 2°C above pre-industrial levels and to pursue further efforts toward a 1.5°C increase limit [5]. To this end, the UNFCCC members have submitted their “Intended Nationally Determined Contributions” (INDCs), in which Parties voluntarily expressed their post-2020 emissions reduction targets. A key aspect of the agreement is the inclusion of a framework for the regular updating of the Nationally Determined Contributions (NDCs) (official INDCs designation after ratification of the agreement) every 5 years to strengthen their level of ambition.

Within this context, Latin America is globally relevant due to: the large share of land-sector emissions (the region accounted for about 20% of global net emissions from Agriculture, Forestry and Other Land Uses (AFOLU) in 2014; [6]); as well as the prospects of growing energy-related emissions in the forthcoming decades [7]. Among the major regional economies, Brazil’s NDC states the commitment to reduce all GHG emissions by 37% in 2025 and 43% in 2030 relative to 2005 levels. Mexico has committed to a reduction of 22% in all GHGs below a business-as-usual (BAU) scenario for the year 2030. Likewise, Argentina has committed to a target of 18% reduction in all GHGs below BAU for 2030 whereas Colombia announced a 20% reduction in all GHGs below BAU for 2030. Regarding the forestry sector, Brazil and Mexico intend to adopt measures to conserve and reforest ecosystems and to reach a rate of zero illegal deforestation by 2030. Along similar lines, Colombia’s NDC indicates a commitment to reduce deforestation and to preserve important natural ecosystems whereas Argentina is planning actions related to the promotion of sustainable forest management. It is worth mentioning that Brazil, which explains the bulk of the regional AFOLU emissions trend, has shown progress by cutting deforestation in the Legal Amazon by 75% between 2004 and 2017 [8]. Nevertheless, recent government acts have weakened environmental control regulations raising serious concerns about a reversal of the deforestation trend [9].

In light of the Latin American NDCs, the understanding of how these pledges can affect the interdependencies among nexus systems is essential to inform coherent policy-making. In this study, we explore potential implications of the Paris pledges on the nexus sectors in Argentina, Brazil, Colombia and Mexico. The analysis is carried out within the framework of the Global Change Assessment Model (GCAM) [10, 11], a state-of-the-art integrated assessment model (IAM), specifically developed to incorporate physical, economic and social domains, and to account for cross-sectoral interactions. The present article builds upon the integrated analytical framework developed to inform specific stakeholders about climate policy-energy-water-land nexus interplays. In this context, we elaborate on an earlier analysis [12] by focusing explicitly on how the nexus asymmetries identified provide suitable instances of the need of cross-sectoral coordination to mainstream the Nexus Approach concept in the overall NDC discussion.

Previous studies have assessed NDCs largely from the point of view of their collective contribution to limit global warming [13, 14], in terms of their implications on the energy sector [15] or on mitigation costs [16, 17]. At the same time, the nexus literature has evolved from a conceptual framework [18] to the recent development and use of analytical approaches to assess and analyze interactions. Albrecht et al. (2018) [19] provides a comprehensive review of the nexus methods found in the peer-reviewed literature that derive from methods used in various disciplines (e.g., integrated modeling, life-cycle assessments, cost-benefit analysis, indicators, among others). Overall, this review identified that quantitative methods to address nexus issues are still limited (less than one third of the literature assessed), revealing a critical need for the development and application of appropriate methods and tools that can support the integrated decision-making. Recognizing that few tools have capabilities to address nexus linkages while allowing the explicit modeling of the Paris pledges, this study relies on a robust self-consistent integrated framework to produce insights unexplored in previous works that assessed NDCs. That is, we explore national level implications of NDCs in the major Latin American economies within a nexus perspective that seeks to highlight the inseparable links between sectors while drawing attention to the emergence of potential macro-scale trade-offs among physical systems. Bearing in mind the close links between the nexus concept and the 2030 Agenda for Sustainable Development [20], in which climate action, energy, water and food securities are pivotal elements, it is therefore becoming clear that nexus trade-offs can undermine the full attainment of the Sustainable Development Goals (SDGs).

The article proceeds as follows. First, we briefly introduce the model and scenarios included in this paper. Next, we present and discuss key potential country-scale implications of the Paris Pledges on the nexus resource systems in the focus countries, examining the relationship with mitigation in the form of two energy-technology options. Then, we conclude by discussing the relevance of the nexus approach applied to the NDCs updating cycles.

## Scenario analysis

### GCAM model

The Global Change Assessment Model (GCAM) simulates the evolution of five key systems (socioeconomics, energy, agriculture and land, water and climate) and their interactions over time. GCAM has contributed significantly to the scientific understanding of climate change as the IAM employed to model the representative concentration pathway (RCP) 4.5 [21] and, more recently, the Shared Socioeconomic Pathway (SSP) 4 storyline [22]. While we focus here on a general description of the essential aspects of GCAM v4.3 relevant for the purposes of the present study, a more comprehensive description of the model is available on the GCAM documentation [23].

Along the first system, socioeconomics, assumptions for population and labor productivity are used to derive GDP in each region, which, in turn, drive the regional economic activity, as well as a large chain of interconnected processes and demand responses in the other systems. Within a market equilibrium economic framework, GCAM represents the global economy by disaggregating the world in 32 geopolitical regions. Latin America and the Caribbean region (henceforward LAC), in particular, is represented as seven distinct regions: Argentina, Brazil, Central America and Caribbean, Colombia, Mexico, South America Northern, and South America Southern (S1 Fig).

As a long-term model, GCAM operates in 5-year time steps until 2100. The base year for the model is 2010, based on calibration to the historical period, which requires multiple datasets for the different GCAM sectors. Further information on the calibration process including data sources can be found as follows: energy [23], agriculture and land [23, 24], climate [13] and water [25, 26]. In terms of solution algorithm, GCAM is a dynamic-recursive model, which solves each period sequentially (based on existing information for the period being solved) through the establishment of market-clearing prices for all existing markets (energy, agriculture, land, GHG emissions). This means that, for each model period, an iterative scheme ensures convergence to final equilibrium prices such that supplies and demands are equal in all markets.

The energy system structure in GCAM contains representations of the energy supply and demand sectors for each region, also considering the trading of primary resources (coal, natural gas, oil and biomass) among regions. The model simulates the temporal evolution of the energy system from the extraction of primary energy resources (oil, natural gas, coal, bioenergy, uranium, hydropower, geothermal, solar, and wind energy) until the transformation processes (e.g., liquid fuel refineries and power generation) that produce the final energy carriers (refined liquids, gas, coal, commercial bioenergy, hydrogen, and electricity) required by the end-use sectors (buildings, industry, and transport). GCAM utilizes a comprehensive technology database that includes more than 100 different energy supply and conversion technology representations and assumptions regarding technological progress [27]. These technologies compete for a share of energy markets based on the relative cost and profit differences [28] such that the model solution represents the lowest-cost and most technically feasible combination of existing technologies and energy resources for each region.

The agriculture and land-use system provides projections of agricultural supply (crops, livestock, forest products, and bioenergy), prices, and changes in land use and cover, taking into consideration the trading of primary agricultural and forest goods. In this component, each of the 32 geopolitical regions can be disaggregated into up to 18 agro-ecological zones resulting in 283 agriculture and land use regions. Within each of these 283 subregions, land is categorized into twelve types based on cover and use (e.g., forestlands, shrublands, grasslands, croplands, etc.). Land allocation within any geopolitical region depends on the relative profitability of all possible land uses within each of the 283 land-use regions [29]. Land used for any purpose competes economically with croplands, commercial forests, pastures, and all lands not involved in commodity production, with the exception of tundra, deserts, and urban lands (assumed constant over time). The profitability of any land used for commercial production is derived from the price (value) of the commodity produced, the costs of production, and the yield [29]. GCAM models the production of twelve crop categories based on exogenously specified yields that are crop-specific but vary depending on the subregion.

Bioenergy production in GCAM derives from: (1) various types of second-generation cellulosic crops (e.g., switchgrass, miscanthus, willow, jatropha, and eucalyptus), (2) residues from forestry and agriculture, (3) municipal solid waste, and (4) traditional bioenergy [23]. Conventional or first-generation biofuel crops such as corn, sugars, oil crops are grown as part of food production. In this case, the biomass liquids subsector within the energy module includes a number of transforming technologies for biofuels production from these food crops. Note that, throughout this analysis, the terms “purpose-grown” and “dedicated” bioenergy feedstocks are used to refer to the second-generation cellulosic bioenergy crops.

The physical atmosphere, oceans and climate are represented in GCAM by the Hector Earth System model [30], which is a reduced-form global climate carbon-cycle model (or simple climate model–SCM). As a SCM, Hector was developed to represent only the most important large-scale earth system processes so that to significantly reduce computational costs relative to the most complex Earth-System Models. Although it can be used as a stand-alone model, Hector is fully integrated within the computational GCAM platform. This coupling allows Hector to track emissions of 24 GHGs and short-lived species generated by the energy, agriculture and land systems and to calculate future GHG concentrations in each modeling scenario. From GHG concentrations and short-lived climate forcers, Hector can then derive global mean radiative forcing, which is converted to global mean temperature and other variables. It is important to note that there is no feedback from Hector on any of the GCAM sectors based on the levels of radiative forcing or global mean temperature achieved from a given emission pathway.

The water module within GCAM provides water demand estimates (gross water withdrawals and net consumptive use) for six sectors: irrigation, livestock, primary energy production and processing, electricity generation, industrial, and municipal. As described by [25, 31], the main characteristics of the GCAM water module are: (1) future agricultural water demands are driven by crop production from GCAM, the share of crop production that takes place on irrigated lands in each of the 283 subregions, and by crop type (12 categories of crops plus biomass). The estimates of water withdrawals for biomass include a number of second-generation biomass crops, but crops such as corn, sugar and oil palm used for biofuel production are not included since their water demands are quantified in the irrigation category. (2) Future manufacturing and domestic water demands are driven by socioeconomic assumptions, among other factors (e.g., total industrial output, future changes in efficiency, technological improvements, and water prices); (3) the water demands for primary energy hinge on the amount of each fuel produced whereas water demands for secondary energy (electricity, refined liquid products) depend also on the specific production technologies used, which in the case of the electric-sector water use includes the types of cooling systems used during thermal power generation.

### Reference and NDC Scenarios

In our model-based scenario approach, we focus on contrasting relevant sectoral outcomes of three scenarios through 2050: the reference scenario and two policy (NDC) scenarios.

The reference scenario is based upon BAU assumptions about key drivers (e.g., population, economic growth and technological evolution), and assumes that no new mitigation actions are implemented beyond 2010. The socioeconomics assumptions are consistent with the “Middle of the Road” SSP 2 [32]. The reference scenario is characterized by population and GDP growth of 26% and 167%, respectively, in LAC from 2010 to 2050 (S2 Fig).

For the two NDC scenarios, the GHG mitigation targets are consistent with the countries’ emissions levels provided in their official NDC submissions [33]. This set of scenarios share the same general assumptions. Nevertheless, they differ with respect to the technology availability in the energy system that is essential to determine how emissions reductions in the energy sector can be fulfilled. The ‘NDC FullTech’ scenario includes the full suite of energy technologies represented by GCAM. On the other hand, the ‘NDC NOCCS’ scenario is based on the explicit assumption that the expansion of CO_2_ capture and geologic storage (CCS) systems is not permitted (all other assumptions are identical to the ‘NDC FullTech’ scenario). New capacities can include nuclear energy in both NDC scenarios. A fundamental motivation for the choice of the technology pathways explored here is that they represent two radically different energy-sector decarbonization routes, each of them with profound consequences for the nexus as a whole. On one side, the ‘NDC FullTech’ scenario allows the opportunity to explore a pathway in which fossil fuel-fired power coupled with CCS, and bioenergy coupled with CCS (BioCCS) become important sources of electricity generation in the long-term. On the other hand, the ‘NDC NOCCS’ scenario is intended to represent a future in which the various limitations surrounding the large-scale deployment of CCS (to be discussed in the following section) could not be overcome and mitigation must rely on other low-carbon sources.

In both policy scenarios, the implementation of the NDCs in GCAM was carried out by means of an economy-wide emissions constraint. This means that the gross GHG emissions (excluding CO_2_ land-use and land-cover change − LUC − emissions) were assigned to each GCAM region and the model internally calculated the carbon prices needed to achieve the constraint. The global GHG emission trajectory follows the ‘Paris-Increased Ambition’ scenario developed in [13] with updates on the emissions constraints for the seven LAC regions. These updates are based upon the supporting sources listed in S1 Text. Note that NDCs only cover the period up to 2030. To allow the exploration of nexus transformations in LAC at a level consistent with the 2°C long-term goal set by the Paris Agreement, it is assumed that beyond 2030 the rest of the world puts forward reduction targets with CO_2_ emissions intensities decreasing at annual rates implied by the NDCs or 5 percent per year, whichever is higher (see [13] for details on these assumptions and the S1 Text for the assumptions in LAC).

It is important to acknowledge that actual climate policy approaches do and will significantly differ from the economy-wide carbon prices approach used herein, relying on a range of different sectoral measures from building standards to automobile fuel efficiency to renewable portfolio standards. The implication for the results in this study is that mitigation is focused more heavily on energy supply adjustments than energy demand changes. For this reason, our results are meant to be purely explorative. However, each NDC scenario encompasses relevant multi-sectoral system-wide interactions that provide useful insights to support the points raised in this paper.

As previously noted, LAC is characterized by a large share of AFOLU emissions compared to the world average. The four countries analyzed in the present study explicitly included the AFOLU sector in their NDCs, however the potential land-based emissions reductions are incorporated within their total reduction targets. As assessed by previous studies [34–36], the NDCs are associated with large uncertainties regarding the actual mitigation role of the land sector. These uncertainties relate to the following issues: definition of baselines, historical emissions and removal sources in national inventories; lack of information on accounting methods; absence of quantifiable details of measures or specific targets, among others. Given that the core of the NDC strategies to curb carbon emissions from the land sector in LAC is formed by forest protection efforts, for the NDC scenarios, we imposed a land-use policy introduced by a carbon tax on LUC emissions from all land types. By penalizing terrestrial carbon emissions, land carbon prices affect the economic decisions within the agricultural/land-use model. As a result, this regime restricts forest conversion to agricultural land and incentivizes forest expansion [11, 37]. Note that despite the implementation of identical terrestrial carbon prices in both scenarios, the land sector responds differently to the interaction with the energy system, leading to different CO_2_ LUC pathways. Finally, terrestrial carbon prices are also applied to all GCAM geopolitical regions to avert the displacement of land-use pressures, i.e., “land-use leakage”, from the four focus countries into those regions.

The emissions pathways (net emissions including CO_2_ LUC emissions) generated by GCAM under the NDC_Full Tech scenario for the four focus regions are shown in Table 1 (see S1 and S2 Tables for the emissions pathways for the remaining scenarios in this study).

**Table 1:** Regional Net GHG emissions (MtCO2_e_) in the NDC_FullTech scenario^a^.

GCAM Region	2010	2020	2030	2040	2050	2030 NDC Target
**Argentina**	736	415	484	488	438	**483**
**Brazil**	2181	1465	1206	1023	825	**1200**
**Colombia**	124	233	266	304	316	**268**
**Mexico**	708	736	759	531	311	**759**

^a^Global Warming Potentials (GWPs) following official NDC submissions. Brazil and Mexico established GWPs from the IPCC Fifth Assessment Report (AR). Argentina and Colombia defined GWPs from the Second AR.

## Results and discussion

### Energy

Although energy-related emissions have been low in Latin America (about 4% of global energy-related CO_2_ emissions in 2015; [38]), the region is expected to face increasing energy demand lined up with its economic development and population growth. In the absence of mitigation, the reference scenario projects a 98% increase in primary energy consumption and a threefold increase in electricity generation between 2010 and 2050, with predominance of fossil fuels and a growing role of natural gas (S3 and S4 Figs). Thus, gross GHG emissions follow a marked upward trend, in particular, Fossil Fuel and Industrial (FFI) CO_2_ emissions, which take larger proportions of the regional emissions up to 2050 (S5 Fig). The curbing of future energy-related emissions is therefore an important mitigation component in LAC’s NDCs. Nevertheless, depending on the available resources and future technology transitions for non-carbon energy sources, substantially different implications on the energy-water-land (EWL) nexus can be expected. Before discussing specific results, it is informative to introduce some of these interplays within a regional perspective.

A first pathway for strong intersections among EWL systems in light of the NDCs is bioenergy. The modern use of bioenergy is recognized as an important tactic to meet part of the future global energy demand while limiting energy-related emissions. The mitigation potential largely increases in the case of BioCCS, which allows the possibility of deep carbon removals and net negative emissions [39–41]. In face of the large-scale bioenergy production necessary for a substantial impact on climate change mitigation (on the order of few hundreds of exajoules (EJ) per year versus present-day levels around 55 EJ; [37]), LAC grows in importance due to its potential for significant increases in production from various feedstock categories [42]. Indeed, LAC is already positioned as a major bioenergy, notably biofuels, producer. Brazil, in particular, has led development for decades focusing on sugarcane products (e.g., bioethanol) that accounted for 17% of domestic energy supply in 2015 [43], not to mention the growing utilization of soybeans for biodiesel production. Biofuels markets also exist in Argentina (e.g., biodiesel from soybeans) and Colombia (e.g., sugarcane ethanol, biodiesel from palm oil), whereas Mexico, one of the largest oil producers in the world, is attempting to develop its biofuels sector hampered by decades of efforts surrounding the petroleum and natural gas sectors [44]. With the approval of the Bioenergy Promotion and Development Law in 2008 and the General Law for Climate Change in 2012 (which sets the goal of 35% of the electricity generated from renewable sources plus nuclear energy by 2024), Mexico aims to increase feedstock production, mainly from agriculture and forestry, also creating opportunities in the agriculture sector [45, 46]. Nevertheless, intensively cropping large areas for dedicated bioenergy production inevitably raises serious concerns surrounding land-use impacts and adverse externalities regarding food and water securities.

Other key nexus interactions unleashed by the NDCs, especially relevant for the energy-water subsystem, stem from an increased participation of low-carbon technologies on the energy system. A larger reliance on renewables such as wind and solar or on CCS technologies involve considerable impacts on the water demands for the electricity sector due to the specific water requirements of each technology. We will further discuss the aforementioned nexus implications in the upcoming sections.

**Fig 1:**
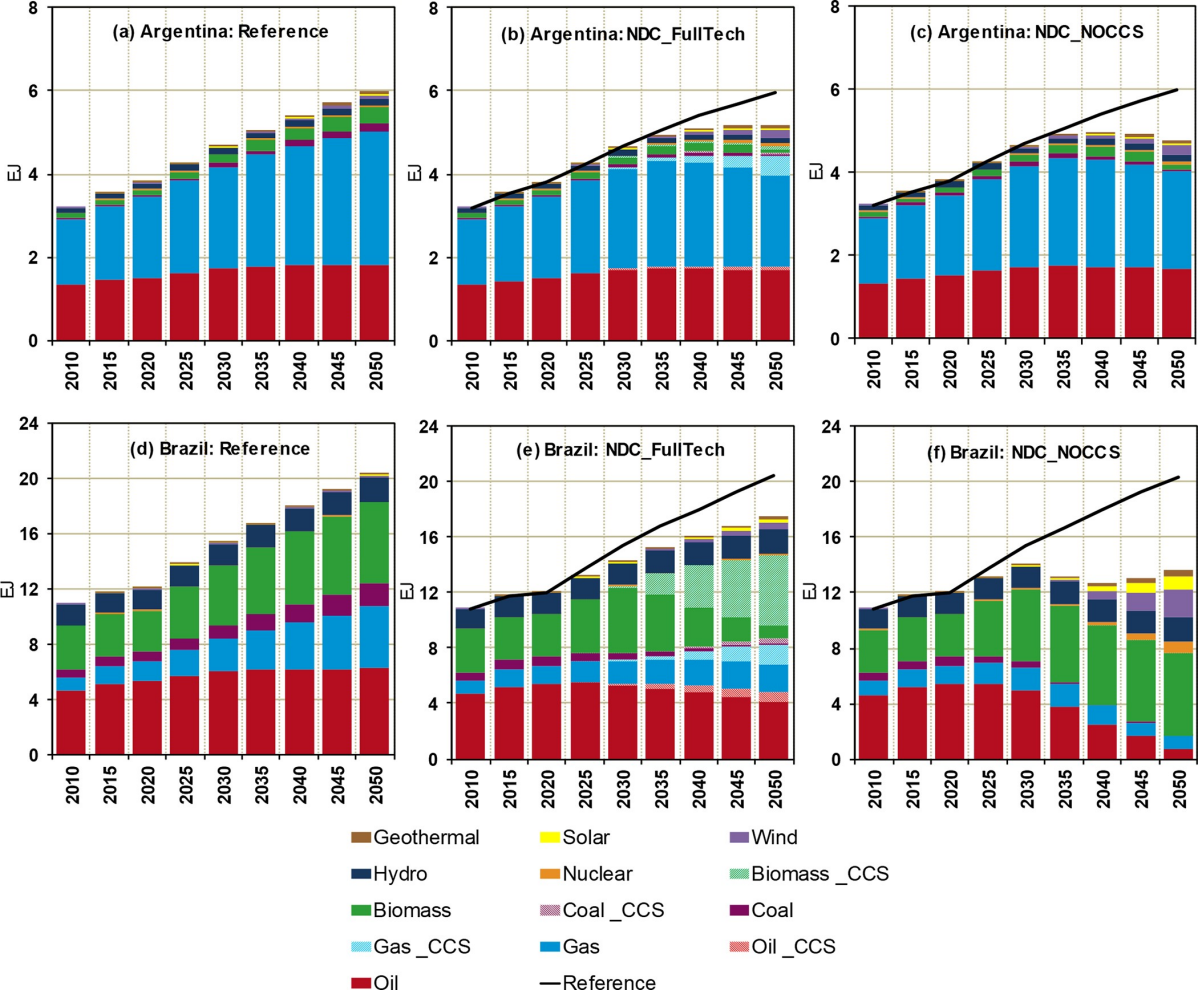
Distribution of the primary energy consumption (EJ) for the Reference ((A) and (D)), NDC_FullTech ((B) and (E)) and NDC_NOCCS ((C) and (F)) scenarios in Argentina and Brazil, respectively.

**Fig 2:**
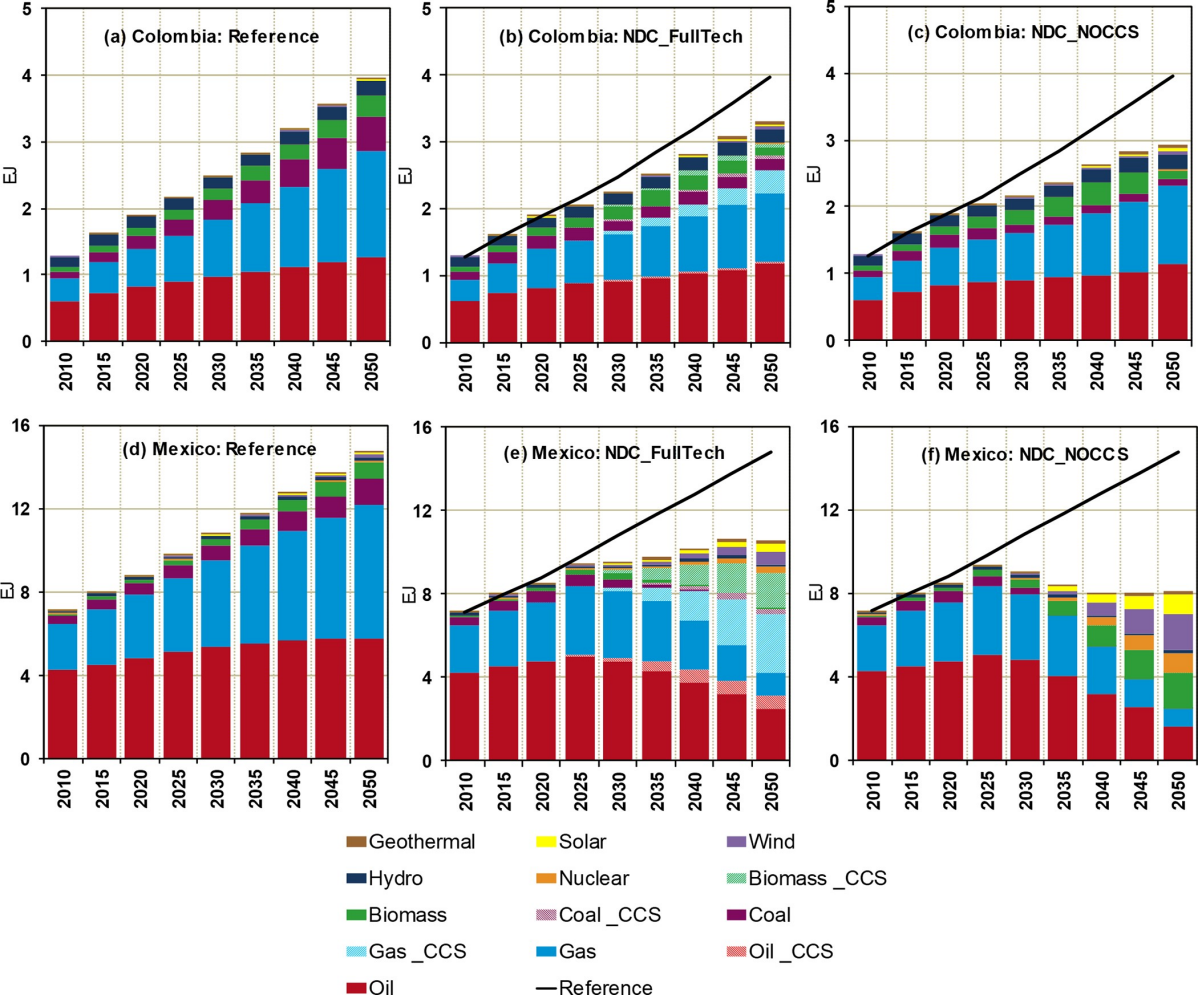
Distribution of the primary energy consumption (EJ) for the Reference ((A) and (D)), NDC_FullTech ((B) and (E)) and NDC_NOCCS ((C) and (F)) scenarios in Colombia, and Mexico, respectively.

As illustrated in Figs 1 and 2, both NDC scenarios entail important transformations of the countries’ energy systems relative to the reference case. These include less fossil-fuel based sources resulting from the larger participation of cleaner energy substitutes in the total primary mix. Carbon prices propagating through energy markets along with the expansion of higher-cost lower-carbon technologies stimulate improvements in the efficiency of energy conversion, driving down demand in all countries up to 2050. This effect is more pronounced in the NDC_NOCCS scenario given the higher energy costs of non-fossil technologies relative to the CCS-coupled options in the NDC_FullTech scenario. In the near-term (2030), changes are relatively small, however, as countries strengthen their mitigation efforts, larger transformations occur over the long-term (2050).

Except for Argentina, when CCS is unavailable (NDC_ NOCCS scenario), biomass plays a larger role in the primary mix relative to the reference scenario. In this scenario, although Brazil accounts for the largest participation of biomass in the primary mix (38% and 44% in 2030 and 2050, respectively), Mexico experiences the largest expansion of biomass consumption relative to the reference with percent increases of 34% and 51% (versus 18% and 3% in Brazil) in 2030 and 2050, respectively. With regards to solar, wind and nuclear energy, the differences between both NDC scenarios are small in the near term, and, overall, these low-carbon sources represent less than 2% of the total primary mix in all countries. Over the long term, the NDC_ NOCCS scenario induces the expansion of solar, wind and nuclear energy, particularly strong in Mexico where these sources represent about 46% of the primary energy mix (versus 4 to 28% in the other countries). This strong development of renewable and nuclear capacities in Mexico is due to the drastic transformations needed within an energy system heavily based on fossil fuels to achieve ambitious long-term reduction goals (50% emissions reduction in 2050 versus 2000, as stipulated in Mexico’s Mid-Century Strategy—see S1 Text). Without CCS as a viable option, significant mitigation by 2050 involves deep structural changes to develop and expand renewable and nuclear capacities. On the other hand, the contribution of these low-carbon options to the primary mix is much lower in the NDC_FullTech scenario (shares of about 12% in Mexico, and 3 to 5% in the remaining countries in 2050) because CCS allows the larger use of fossil fuels.

Under the NDC_FullTech scenario, the largest expansion of CCS occurs in Mexico followed by Brazil, reflecting the scale of their energy systems and the amount of mitigation needed in each country. On the other hand, Argentina shows the lowest level of CCS development. BioCCS significantly expands in Brazil whereas the remaining countries develop more natural gas with CCS than BioCCS over the long term. Although the large-scale deployment of CCS is widely accepted as a key strategy to achieve deep CO_2_ emissions reductions over the long-term and to reduce the associated mitigation costs, the viability of such approach is still highly uncertain. Globally, CCS technologies have not yet been broadly deployed commercially. This is due to barriers such as the significant research & development investments required to overcome the technological challenges involved in their safe and cost-efficient utilization, or even the lack of political and policy support [47, 48]. In the particular context of LAC, some authors argue that CCS capabilities could be a less viable option compared to other countries for reasons that include lack of major technological and institutional development [49, 50]. Despite a mature technology, similar arguments hold for nuclear energy when referring to its future viability as a low cost option for mitigation in LAC. Presently, the level of nuclear electricity generation in LAC is low. Nuclear energy in Argentina, Brazil and Mexico accounted for 6.9, 2.9 and 4.8% of their total electricity in 2009 [51], respectively (there is currently no nuclear power plant in Colombia). Over the short-term, there is limited growth prospects in nuclear capacity in these countries since only 1 nuclear plant is under construction in Argentina and Brazil. High operational and investment costs, need of foreign technical expertise and public resistance have slowed down the expansion of nuclear energy in LAC and may prove to be significant obstacles to hamper its future expansion in the region relative to renewables. In this paper, we do not take up the question of how likely CCS and nuclear energy are to become viable options for future implementation in LAC, but rather focus on the understanding of their potential nexus implications.

### Land

Favored by its vast swathes of productive land, LAC almost tripled its net food production since the early eighties, becoming a major food exporter [52]. In the present context of globalized food systems [53], LAC accounts for 38% of oil crops, 30% of fruits and 19% of meat global exports [54]. This process, spurred by a monumental global demand growth for agricultural commodities (e.g., soybeans, meat, and tropical timber), has induced extensive clearing and degradation of native forests, savannas (e.g., the “Cerrado” in Brazil), shrublands and grasslands (e.g., the “Pampas” in Argentina), with South American countries, notably Brazil and Argentina, playing major roles [55].

Notwithstanding the fact that most of the projected growth in crop production should derive from higher yields and increased cropping intensity, Latin America in tandem with the sub-Saharan Africa are expected to account for the bulk of future global agricultural land expansion [56]. The largest tracts of land with rainfed crop production potential are concentrated in Brazil followed, in LAC, by Argentina, Colombia, Bolivia, Venezuela and Peru [57]. Nevertheless, such a vast fertile territory is not entirely available for agricultural expansion since it encompasses sensitive ecosystems, protected areas and urban zones. For instance, legally protected reserves and indigenous territories represented 47% of the Brazilian Amazon region in 2012 [58], with increasing efforts toward forest and land protection regulations in other LAC countries as well [59]. It is widely agreed that the conversion of such areas into crop or pastoral land implies enormous economic and social costs alongside environmental impacts inconsistent with the land-sector mitigation efforts necessary for climate change stabilization.

Even though deforestation in LAC is a rather complex system of many drivers operating simultaneously, land-energy interplays are also critically relevant, as exemplified by the existing logging for fuelwood and charcoal production, and the expansion of hydropower capacity in the Amazon basin [60]. In addition, land-energy nexus considerations are central to the bioenergy debate in LAC given the prospects of continued increase in internal and external market demand for biofuels. Several studies have shown that the large-scale cultivation of bioenergy crops, unless produced from abandoned agricultural or marginal lands, could exacerbate land competition inducing: (1) loss of undisturbed ecosystems (which, in turn, increases LUC emissions that offset the intended mitigation benefits) and biodiversity stocks [61–63]; and (2) displacement of farmland that contributes to drive up food prices [11, 37, 64]. Although the most controversial debate on the impacts to the land sector are around the first-generation bioenergy (food) crops [65], the second-generation bioenergy can potentially unleash additional impacts if supplied by dedicated plantations [66].

**Fig 3:**
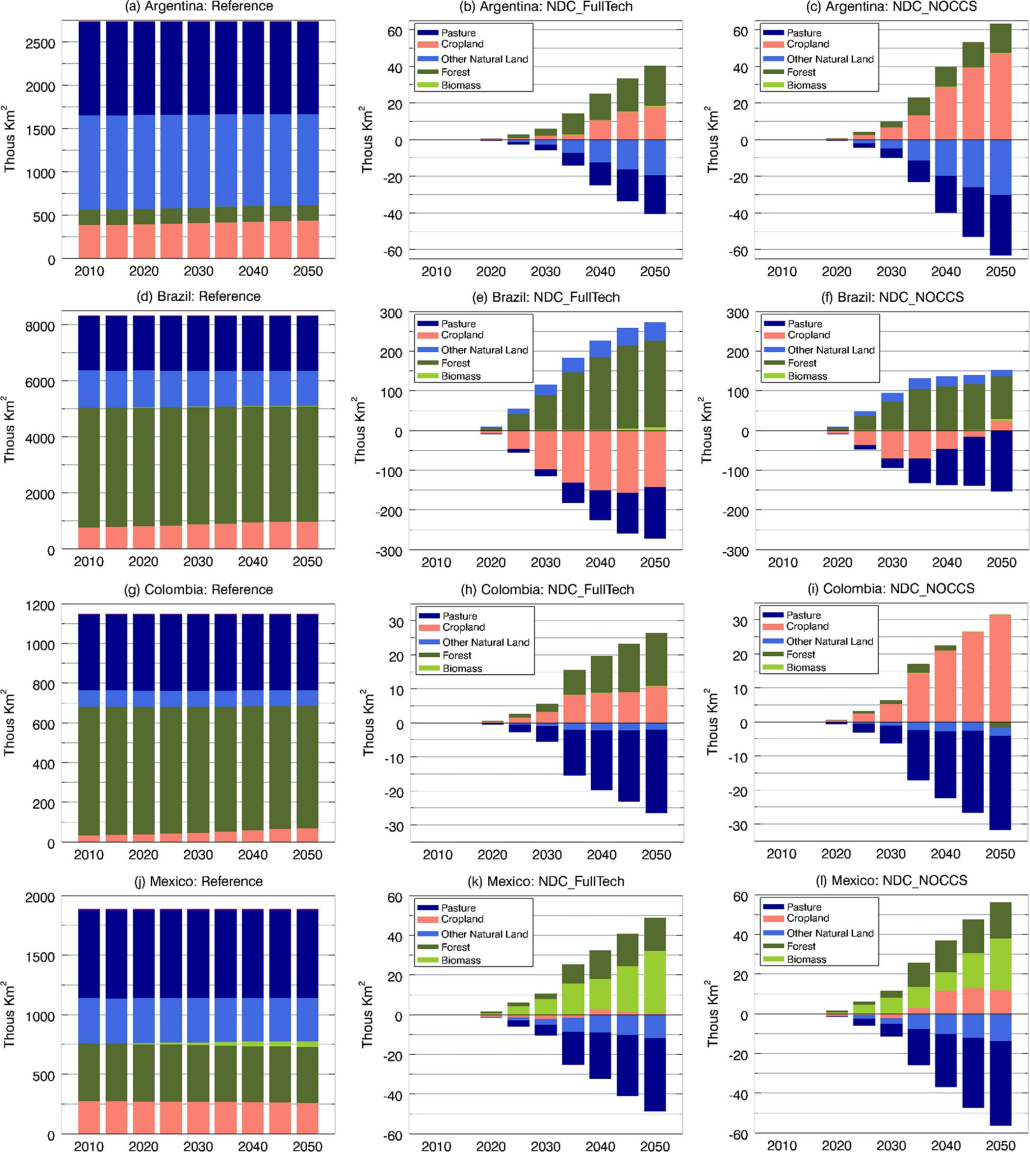
Land allocation (thous. Km^2^) under the Reference ((A), (D), (G), and (J)). Difference in land allocation between the NDC_FullTech and the reference pathways in (B) Argentina, (B) Brazil, (H) Colombia, and (K) Mexico. Difference in land allocation between the NDC_NOCCS and the reference pathways in (C) Argentina, (F) Brazil, (I) Colombia, and (L) Mexico.

The picture emerged from the above discussion is that, even within a context of relatively land-abundance, future land use and availability in LAC is subjected to various conflicting demands that can be affected by NDCs. In this context, two relevant development NDC modes can be distinguished: (i) increased bioenergy production to accommodate the internal demand for low-carbon sources and exports to regions with limited land and/or feedstock resources, and (ii) stringent actions to conserve and restore natural forests and ecosystems. Fig 3 explores these modes by showing the projected distribution of the land use in the four analyzed countries under the two NDC scenarios. Focusing on the differences between the reference (left panels of Fig 3) and the NDC scenarios, it can be noted that relative changes in land cover associated with dedicated biomass production tend to be pronounced in Mexico. This is due to the cost-efficiency of this option given the amount of mitigation required to transform the emissions baseline profile in Mexico that comprises the largest share of CO_2_ FFI emissions among the four focus countries.

In Brazil, the proportional growth in land for bioenergy crop production in both NDC scenarios is far less important than the changes in other land uses (Fig 3D–3F). This may seem counter-intuitive considering the current prominent role of Brazil in the bioenergy sector. Referring back to results from the previous section, we have noted that biomass consumption in the Brazilian primary energy mix under both NDC scenarios is not projected to substantially increase compared with the reference (that already relies on large bioenergy usage). Furthermore, the NDC scenarios include pricing of terrestrial carbon that incurs high economic costs for the large-scale clearing of the carbon-rich forested systems. Since the expansion of land to grow dedicated bioenergy crops is an uneconomic option under the NDC scenarios, the emissions reduction required by the Brazilian NDC needs to be achieved by other low-carbon means. Finally, in Argentina and Colombia, dedicated bioenergy crop production is not projected as a major source of land-use pressure under the NDC scenarios.

Fig 3 also reveals that forests expand throughout the 2030–2050 horizon in all countries under both NDC scenarios. The largest increments are projected for Brazil at the expense of croplands and pasture. As croplands become more profitable, GCAM projects an expansion of croplands into pasturelands and lands dedicated to other natural systems (e.g., shrublands, savannahs, grasslands, etc.) in both NDC simulations in Argentina and Colombia, particularly in the NDC_NOCCS scenario. In Mexico, the long-term expansion of croplands is proportionally less pronounced because of the pressure for land to increase bioenergy production.

### Water

LAC is endowed with impressive 32% of the global renewable water resources [67]. Despite the overall abundance, water resources are unevenly distributed throughout the region. For instance, Mexico and Argentina experience water deficits, particularly in the northern Mexico and some parts of Argentina where moderate to severe water scarcity conditions last more than six months [68]. In fact, the northern and central areas of Mexico, that concentrate 77% of the population and 87% of GDP, constitute prominent examples of low natural availability aggravated by overconsumption of freshwater resources. They also serve to call immediate attention for the fact that water supplies are expected to be placed under increasing stress from socioeconomic trends whose signal can outweigh the effects of climate change in the near future [69].

**Fig 4:**
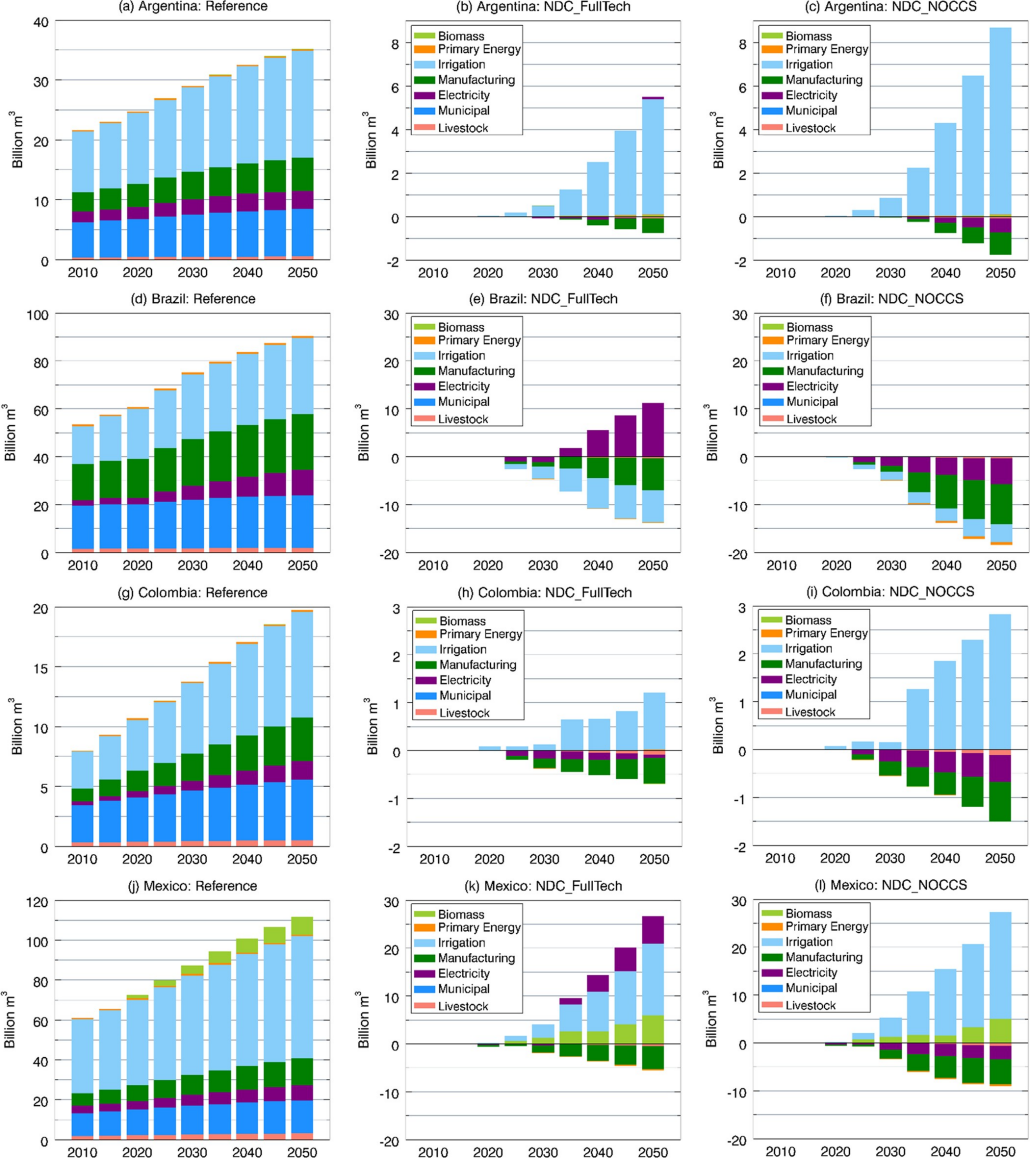
Total water withdrawals by sector (billion m^3^) under the Reference ((A), (D), (G), and (G)). Water withdrawal differences between the NDC_FullTech and the reference pathways in (B) Argentina, (E) Brazil, (H) Colombia, and (K) Mexico. Water withdrawal differences between the NDC_NOCCS and the reference pathways in (C) Argentina, (F) Brazil, (I) Colombia, and (L) Mexico.

Under the BAU assumptions of the reference scenario, the water demand for different uses, particularly agricultural irrigation, increases at alarming rates over the coming decades (S6 Fig). Mexico is the main water user, followed by Brazil (see Fig 4(D) and 4(J)). This is mainly due to the role of irrigated agriculture in Mexico, which currently has the largest area of irrigated land in LAC (about 6.5 million ha) with an infrastructure based predominantly on water-inefficient surface (flood) irrigation techniques. In this regards, Brazil and Argentina also maintain sizable areas of land equipped for irrigation with 2.9 and 2.4 million ha, respectively [67]. Also important within this context is the fact that Latin America is characterized by an overall low irrigation efficiency—average of 39% (the highest efficiency is in Brazil with 41%)—contrasting to the global average of 56% [70].

Previous discussion highlights potential implications of NDCs in terms of land-cover change driven by the need to grow dedicated bioenergy crops. Likewise, impacts on the water use can be expected. Water requirements for bioenergy crops vary considerably with crop type, climate and soil conditions, but, in general, bioenergy derived from agricultural feedstocks is more water intensive than fossil fuels, particularly in the case of first-generation biofuels [1, 65, 71]. Certain second-generation bioenergy crops have disadvantageous water footprints as well [72–74].

Lastly, NDCs may also imply drawbacks related to the water use for power generation. In this sector, LAC is characterized by heavy use of hydropower generation (see S6 Fig), particularly notable in Brazil and Colombia. This option entails significantly lower water consumption (basically due to evaporation losses) than other power generation sources. Although some growth is expected through mid-century, the share of hydropower in the electricity mix should decrease over time due to limitations on natural resources availability. Hence, other power generation sources will have to increase participation in the electricity mix to account for the escalating demand in the region. This means that LAC will potentially need to deal with challenges posed by the larger water requirements of conventional thermal power plants. These challenges may be exacerbated by an NDC climate policy if mitigation of energy-related emissions focuses heavily on CCS. Compared with conventional thermal power plants, CCS-based power plants generally have higher water requirements due to additional demands for cooling and other processes that increase water consumption by 37–95% depending on the power plant type [75, 76]. On the other hand, climate mitigation through solar photovoltaic (PV) and wind is not water intensive. In operational solar facilities, some water is needed to clean the mirrors/panels. However, concentrating solar power (CSP) systems can be considered as water-intensive as traditional thermoelectric power plants because of the additional water usage for cooling processes that is maximized if wet-cooling methods are employed [77]. The aforementioned differences in water usage between thermal (with or without CCS) and non-thermal renewable types of energy supply are detailed in Table 2. Data are presented in terms of median values of the ranges of water consumption and withdrawal factors compiled by [78]. These values are used within the methodology described by [79] to specify GCAM water use intensities by electric-sector technologies. Table 2 makes clear the fact that a transition toward a less carbon-intensive power sector (through nuclear, CCS or CSP facilities) may result in an increase in total water usage depending on the combination of sources, cooling systems and technologies employed.

**Table 2:** Water Use Factors for Electricity Generating Technologies (gal/MWh).

Fuel Type	Cooling	Technology	Median Values	
			Consumption	Withdrawal
Nuclear	Tower	Generic	672	1101
	Once-through	Generic	269	44350
	Pond	Generic	610	7050
Natural Gas	Tower	Combined Cycle	198	253
		Steam	826	1203
		Combined Cycle with CCS	378	496
	Once-through	Combined Cycle	100	11380
		Steam	240	35000
	Pond	Combined Cycle	240	5950
	Dry	Combined Cycle	2	2
	Inlet	Steam	340	425
Coal	Tower	Generic	687	1005
		Subcritical	471	531
		Supercritical	493	609
		IGCC	372	390
		Subcritical with CCS	942	1277
		Supercritical with CCS	846	1123
		IGCC with CCS	540	586
	Once-through	Generic	250	36350
		Subcritical	113	27088
		Supercritical	103	22590
	Pond	Generic	545	12225
		Subcritical	779	17914
		Supercritical	42	15046
BioPower	Tower	Steam	235–553	878
	Once-through	Steam	300	35000
	Pond	Steam	390	450
PV	N/A	Utility Scale PV	26	i
Wind	N/A	Wind Turbine	0	
CSP	Tower	Trough	865	
		Power Tower	786	
		Fresnel	1000	
	Dry	Trough	78	
		Power Tower	26	
	Hybrid	Trough	338	
		Power Tower	170	
	N/A	Stirling	5	

i Withdrawal factors assumed to be equivalent to consumption factors.

To provide a perspective on the potential implications of the Paris pledges on national water demands in the focus LAC countries, Fig 4 disaggregates differences by sector in water withdrawal estimates between each NDC scenario and the reference. Note that the water demands estimated by GCAM are not constrained in terms of future water supplies, and that climate change impacts are not included. Under both NDC scenarios, the overall picture across the countries, except for Brazil, is one of larger water footprint in a growing pattern until the midcentury. Fig 4 also brings out the fact that crop irrigation accounts for great part of the increments in total water withdrawals. Brazil is the only country where the near and long-term total water demands are projected to decline under both NDC scenarios, as well as the specific demand for irrigation (Fig 4D–4F).

**Fig 5:**
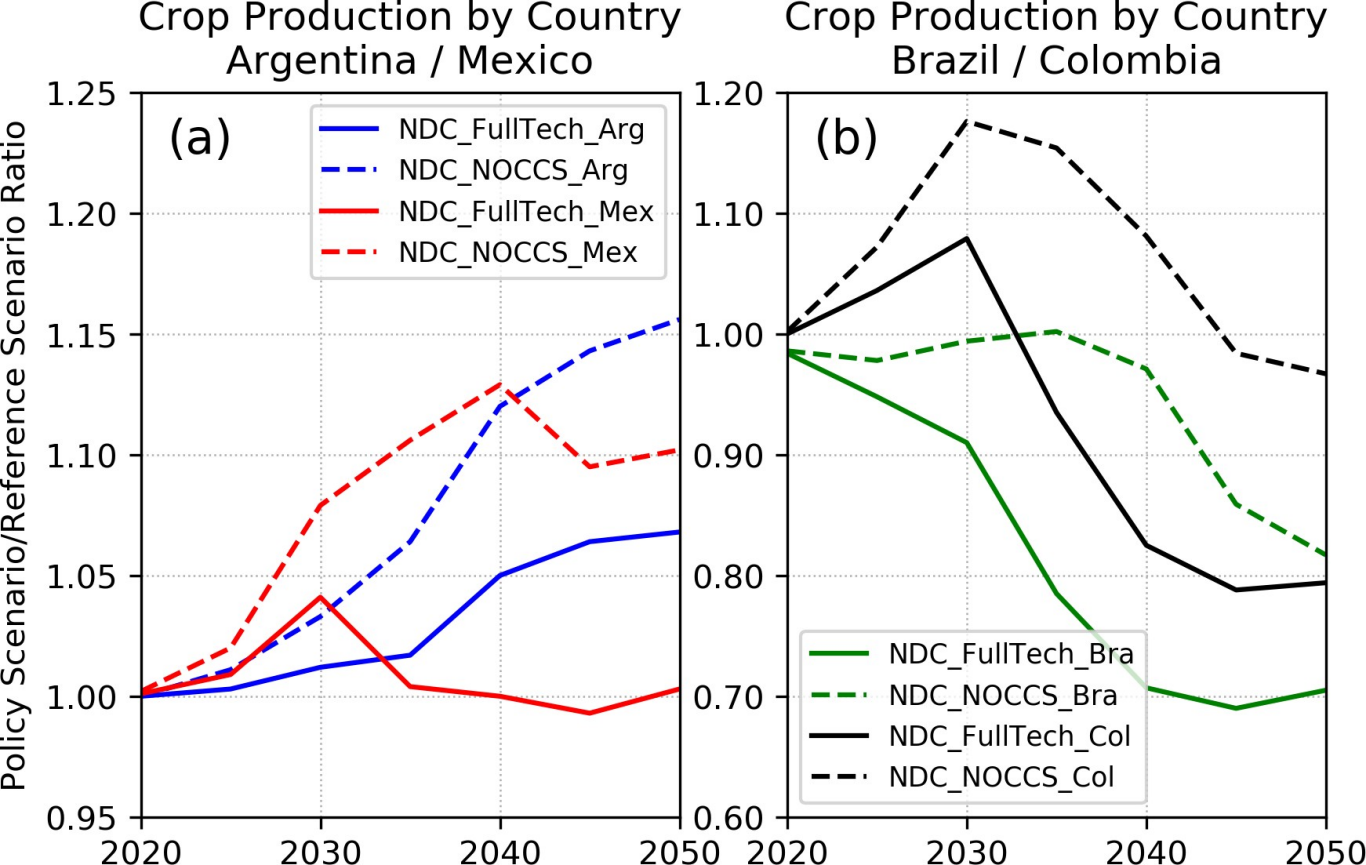
Crop production by country expressed as the ratio between each NDC scenario and the reference scenario. For each country, the amount of agricultural production only includes those crop categories in which a share of their total production is under irrigation. This means that the amount of crop production accounted in this figure does not reflect the total crop production calculated by GCAM for each country.

The previous results point to relevant interactions between the water and land sectors derived from shifts in the amount of land available for crop production, which, affected agricultural production in each region and scenario. For example, in Argentina, the pressure to expand croplands in both NDC scenarios (recall Fig 3) allowed increased crop production (Fig 5), leading to higher irrigation demands, whereas, in Brazil, the opposite is verified.

Regarding irrigation for bioenergy crops, considerable demands are seen in Mexico under both NDC runs in the near and long term (Fig 4J–4L). This is in line with results from previous sections that point out Mexico as the country with the largest proportional increase in biomass participation in the energy mix and the most relevant land-cover expansion to grow bioenergy feedstocks. In general, both NDC scenarios are associated with less water being demanded by the manufacturing sector over time. This is attributable to commensurate declines in the total industrial output in the NDC scenarios that is the main driver for future water demands in the GCAM manufacturing sector [25].

Across all countries, changes in the electric sector water withdrawals can be noted in both NDC scenarios in response to the availability of CCS. In general, both NDC scenarios signal modest water withdrawals reductions relative to the reference in the near term, which are due to small reductions in electricity generation and the consequent less use of water in the power generation process. When CCS is available (NDC_FullTech scenario), Brazil and Mexico show larger water withdrawals over the long term, consistent with the timeframe when CCS is substantially deployed in these countries. On the other hand, lower water demands are observed in Colombia throughout the simulation period. Given the modest level of CCS deployment in the Colombian primary mix (shares of 1% and 14% in 2030 and 2050, respectively), lower electricity generation and reduced thermal power water demands (compared with the reference case) played more relevant roles. In Argentina, which shows the lowest level of CCS deployment, no major water demand pressure in the power sector is noted.

**Fig 6:**
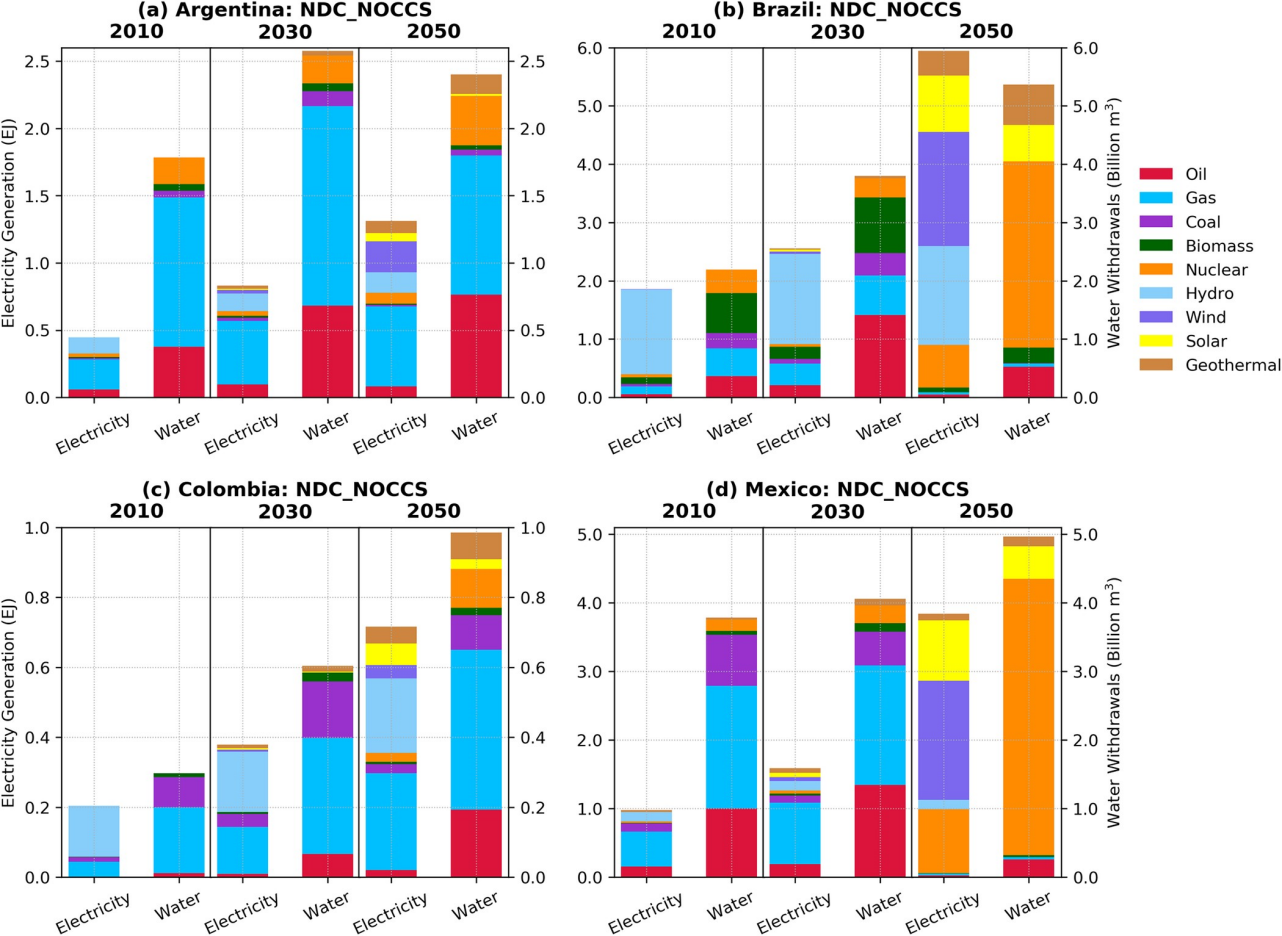
Water withdrawals (right bars) by power generation source (left bars) under the NDC_NOCCS scenario for (A) Argentina, (B) Brazil, (C) Colombia and (D) Mexico.

In the case of the NDC_NOCCS scenario, the water demands for electricity are consistently lower than the reference in all countries. While in the near-term, this is mostly due to a reduction in power generation, the overall long-term reduction in water withdrawal volumes results from the expansion of wind generation and solar-powered electricity (Fig 6), even considering the water-intensive nuclear energy, which also increases participation in this scenario (recall Figs 1 and 2). Note that the long-term expansion of solar energy displayed in Fig 6 also includes the water-intensive CSP systems that respond for most of the water withdrawal volumes associated with solar energy in 2050. However, the overall net effect of the expansion of renewables in the NDC_NOCCS scenario is to reduce power-generation water demands.

## Conclusions

This study presents an integrated assessment of potential implications of mitigation strategies consistent with the Paris Agreement architecture on the EWL nexus resource systems in Latin America. By means of the GCAM modeling framework, we have developed mitigation scenarios in which targets are consistent with the NDCs submitted by Argentina, Brazil, Colombia and Mexico, followed by stringent post-2030 emissions reductions assumptions. The two policy scenarios explored herein were characterized by differing degrees of low-carbon technology deployments in tandem with a land-sector strategy that prevented forest loss and stimulated afforestation. This approach allowed the opportunity to explore two radically different energy-sector decarbonization routes and their interplays with the land and water sectors in each country. Based on our partial equilibrium modeling, we find that the policy scenario results entail relevant differences relative to a baseline case. Those can be summarized as follows: (1) growing irrigation demands up to the midcentury in all countries, except for Brazil; (2) larger irrigation demands to cultivate bioenergy crops in Mexico; and (3) larger electric-sector water withdrawals in countries that largely deploy CCS over the long-term (Mexico and Brazil) versus reduced demands when CCS is unavailable.

Given the scenario outcomes, the central insight of our study is that the implementation of NDCs in LAC can result in critical country-level synergies and trade-offs within the nexus domain associated with the portfolio of mitigation strategies in place. Relevant consequences of mitigation can be unleashed in many ways. One important factor is the range of forest protection measures (a crucial mitigation component in Latin America), which affects the overall cropland availability. This process, in turn, may interfere with food production levels and irrigation demands. For example, in Brazil where our results revealed forested areas growth partially achieved at the expenses of croplands, there were implications in terms of reduced food production and lower irrigation demands. In addition, the results from Argentina, Colombia and Mexico suggest that non-forested ecosystems, most of them already under serious threats, may be put at additional pressure within a land-sector mitigation framework centered on forest protection. As shown by [80], within a forest conservation scheme, such areas become major options for cropland expansion, thus requiring efficient land management and technological innovations in agriculture for their protection. Within the land and water domains, results from Mexico call for careful consideration on the role of the second-generation bioenergy in future mitigation strategies in face of the land and water requirements to cultivate bioenergy crops. Finally, the role a transition toward a less carbon-intensive power sector may play in increasing electric-sector water usage in LAC was made clear in the results. As previously discussed, low-carbon sources with high water requirements are CCS, which we have emphasized in our scenario design, but also CSP and nuclear energy.

In face of a potential trade-off between agricultural water demands and the climate policy, our results highlight the need of demand-side responses that incorporate improvements in water and land management with coordinated actions. Options applicable to the arid and semi-arid regions of Latin America include increased irrigation efficiency and changing cropping patterns toward less water-intensive and drought-resistant crops as well as no-tillage practices [81, 82]. Given the inefficient irrigation infrastructure in LAC, which is heavily reliant on surface (flood) irrigation (95.6% of irrigated lands; [83]), important water savings could result from the implementation of modern irrigation methods. Mean differences in field application efficiencies between the least efficient surface systems and the sprinkler and drip systems are about 27 and 40%, respectively, in the South America region [84]. These large differences suggest that the additional agricultural water demands found in our results could be reduced by a shift in irrigation technology. Although undoubtedly beneficial from the water perspective, advanced management practices in water and land may be difficult to implement in practice due to various factors such as their higher investment and maintenance costs, lack of efficient institutions and need of policy incentives. Moreover, there are potential trade-offs associated with increased on-farm energy demands and GHG emissions [85] that need to be accounted for.

Circumventing climate change through ambitious efforts is the major priority within the Paris Agreement framework. In this context, the full implementation of the current NDCs has been related to important reductions of the post-2020 GHG emissions. Nevertheless, these emission pledges have been considered insufficient to limit global warming to less than 2<C without a substantial enhancement of global mitigation efforts after 2030 [13, 14]. Ramping-up the stringency of the Paris pledges will be a focus of attention in the coming years as Parties are requested to resubmit their NDCs by 2020, and periodically assess their progress by means of a process known as global stocktake (first global stocktake planned for 2023). To inform the global stocktake process, a number of studies [86–88] have pointed out the necessity of a systematic and broader process of assessment of the progress of the goals of the Paris Agreement through a multi-objective framework that incorporates, for example, the implications of NDCs on the SDGs.

Our study then reveals relevant implications for the aforementioned deliberations that will support the updating and enhancing of the NDCs. First, our post-2030 results highlight the potential exacerbation of cross-sectoral implications in the four major LAC economies when mitigation efforts are strengthened. Hence, more ambitious NDCs may imply higher risks of unintended consequences (see further comments on the potential exacerbation of mitigation trade-offs under stringent climate targets below). Second, the clear common objectives within the nexus concept and in the set of goals and targets of the SDGs reinforce the value of an assessment and updating NDC framework that incorporates considerations on the nexus sectors and their interdependencies as a mean to contribute toward sustainable development.

While this study provides important insights regarding the climate policy (NDCs)-energy-water-land nexus interplays in LAC, any conclusions drawn should be mindful of the assumptions underlying the model and scenarios. For example, technology availability is a critical assumption in our study. In this respect, one could argue about the relatively high long-term deployment of nuclear energy in Brazil and, particularly, in Mexico under the NDC NOCCS scenario, which may seem a high deployment pathway compared with the current role of nuclear energy in LAC. We find that limiting future nuclear energy expansion in LAC does not affect the broad nature of the key trade-offs and synergies previously discussed, although power-sector water savings might potentially increase under a combination of CCS unavailability and constrained nuclear capacity (further description on this supplementary analysis focused on the role of nuclear energy can be found in S2 Text). A limitation in terms of technology relates to the fact that GCAM currently does not have explicit representations of the various existing irrigation systems, which would be important to guide relevant decision-making in LAC. It is also worth mentioning that GCAM water delivery-efficiency factors, assigned by crop type and region, are held constant over time [25, 26]. An additional aspect of our modeling approach is that water supply is assumed an unlimited resource. This means that our study does not incorporate feedbacks exerted by physical water constraints from growing regional demands or climate change on energy and agricultural systems. In fact, climate change can result in additional pressure on nexus systems in LAC. This type of concern has been supported by robust differences in regional climate characteristics between present-day and global warming of 1.5°C and between 1.5°C and 2°C warming levels [89]. Future research should then be directed at incorporating climate impacts on the water supply as well as on the renewable energy potential to understand how such stressors will propagate across the nexus systems in LAC. Moreover, land policies influence the amount of mitigation effort needed in the energy sector, also interfering with land availability for food production. Hence, our results are sensitive to the land-policy (implemented via terrestrial carbon-prices) applied here. Additional steps toward a better understanding of the implications of climate policies on the EWL nexus in LAC will require the implementation of comprehensive land-related policies, which will reveal important interplays with the other sectors.

Finally, it is important to note that our analysis focuses on the upper bound of the Paris Agreement long-term climate goals in line with previous literature that has examined 2°C-compatible scenarios (e.g., [16]). Nevertheless, the Agreement called for additional efforts to limit end-of-century global warming to 1.5°C above pre-industrial levels in order to minimize damaging climate change impacts [5]. Previous global-scale studies [90, 91] that have examined differences between 1.5°C and 2°C scenarios emphasized that the 1.5°C target requires faster decarbonization of the energy supply, CO_2_ neutrality around the mid-century, net negative emissions in the 2050–2100 period, greater efficiency and demand-side reductions and profound transformations in the land-use. Hence, increasing mitigation ambition from 2.0 ^o^C to 1.5 ^o^C may result in greater and no-trivial challenges within the nexus in Latin America. Moreover, the manner in which emission reduction policies are implemented can lead to different pathways in terms of nexus synergies and trade-offs. As shown by [91], increasing mitigation ambition from 2 ^o^C to 1.5 ^o^C in scenarios characterized by economy-wide policies implemented via global cost-minimizing carbon prices exacerbated trade-offs such as those associated with land requirements for bioenergy, CCS and water extraction. On the other hand, when a range of sustainable policy measures were incorporated into the original policy design, mitigation risks could be largely alleviated or even compensated. Further research is then needed to examine the implications for the EWL nexus in LAC of the transformations required to meet the 1.5 ^o^C goal to address, for example, the possibility of exacerbation of nexus trade-offs relative to the 2 ^o^C warming level and under which policy mechanisms new stresses or synergies can emerge.

The results and insights outlined above offer an opportunity to discuss a change in the manner current decision-making has been made about NDCs, that is, without sectoral integration and strategic planning to minimize potential nexus trade-offs. Embedding the ‘Nexus Approach’ in the policy debate regarding NDCs is critical to align a more efficient stewardship of nexus resources with NDCs progressively more stringent with time.

## Supporting information

S1 Fig GCAM representation of the 7 Latin American regions. (PDF)

S2 Fig Socioeconomics assumptions for the scenarios developed in this study. (PDF)

S3 Fig GCAM outputs for the Reference (no policy) scenario: Primary energy consumption (EJ) by region (top) and by source (bottom).
(PDF)

S4 Fig w GCAM outputs for the Reference (no policy) scenario: Primary energy consumption (EJ) by region (top) and by source (bottom).
(PDF)

S5 Fig GCAM outputs for the Reference (no policy) scenario: Greenhouse gas emissions (MtCO_2_e; excluding CO_2_ LUC emissions) by region (top) and by source (bottom).
(PDF)

S6 Fig GCAM outputs for the Reference (no policy) scenario: Total water withdrawals (Billion m^3^) by region (top) and by source (bottom).
(PDF)

S1 Table Regional Net GHG emissions (MtCO_2_e): Reference Scenario. (DOCX)

S2 Table Regional Net GHG emissions (MtCO_2_e): NDC_NOCCS Scenario. (DOCX)

S1 Text Assumptions about emissions constraints in the Latin America and the Caribbean Region in the NDC Policy Scenarios. (DOCX)

S2 Text Main results from the ancillary NDC Policy Scenarios: Long-Term limited nuclear energy capacity in Latin America. (DOCX)
