# Supplementary material for: The Paris pledges and the energy-water-land nexus in Latin America: Exploring implications of greenhouse gas emission reductions
Source: PLoS One. 2019 Apr 16;14(4):e0215013. doi: 10.1371/journal.pone.0215013 (PMC6467372; doi:10.1371/journal.pone.0215013)
Supplement: S1 Fig — (PDF) [file pone.0215013.s001.pdf]

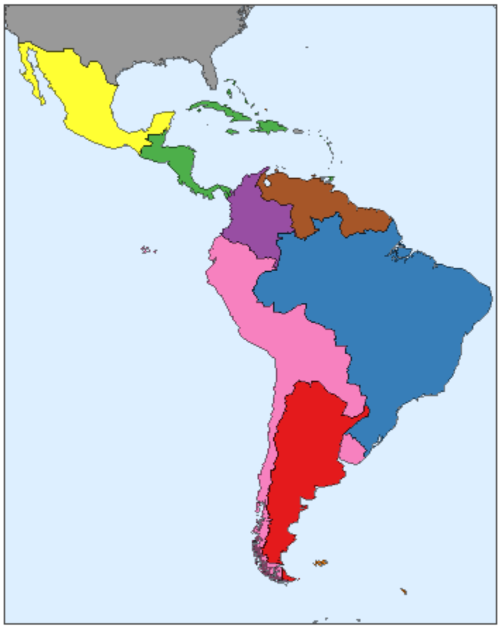

GCAM representation of the 7 Latin American regions: Argentina (red), Brazil (blue), Central America and Caribbean (green), Colombia (purple), Mexico (yellow), South America Northern (brown), and South America Southern (magenta).
