# Supplementary material for: The Paris pledges and the energy-water-land nexus in Latin America: Exploring implications of greenhouse gas emission reductions
Source: PLoS One. 2019 Apr 16;14(4):e0215013. doi: 10.1371/journal.pone.0215013 (PMC6467372; doi:10.1371/journal.pone.0215013)
Supplement: S2 Fig — (PDF) [file pone.0215013.s002.pdf]

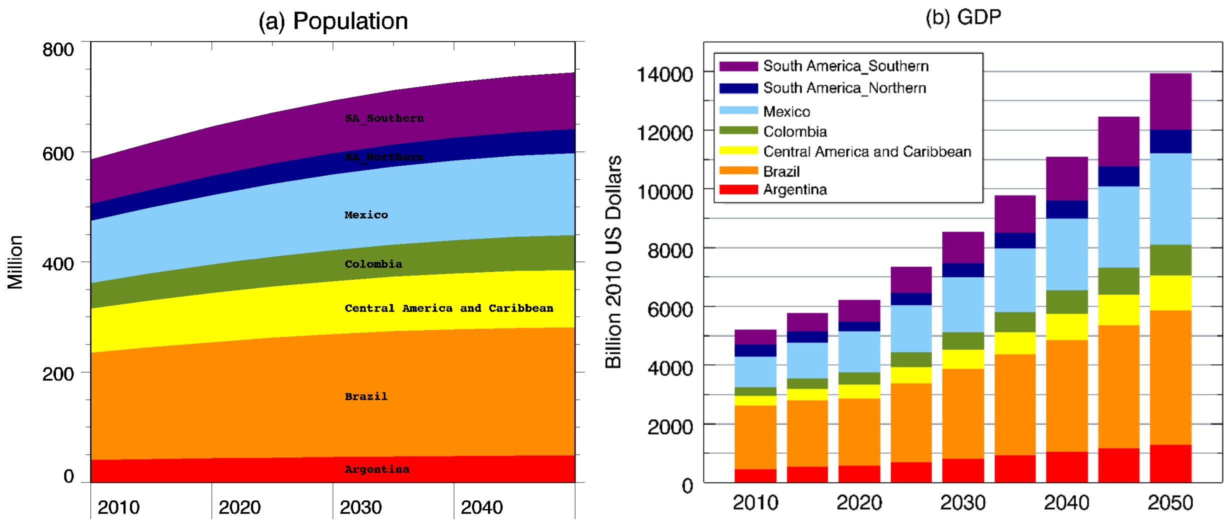

Socioeconomics assumptions for the scenarios developed in this study: (a) Population and (b) Gross Domestic Product (GDP). Short-term GDP: IMF World Energy outlook database. Long-term GDP and Population: Shared Socioeconomic Pathways database (SSP2).
