# Supplementary material for: The Paris pledges and the energy-water-land nexus in Latin America: Exploring implications of greenhouse gas emission reductions
Source: PLoS One. 2019 Apr 16;14(4):e0215013. doi: 10.1371/journal.pone.0215013 (PMC6467372; doi:10.1371/journal.pone.0215013)
Supplement: S4 Fig — GCAM outputs for the Reference (no policy) scenario: Primary energy consumption (EJ) by region (top) and by source (bottom). (PDF) [file pone.0215013.s004.pdf]

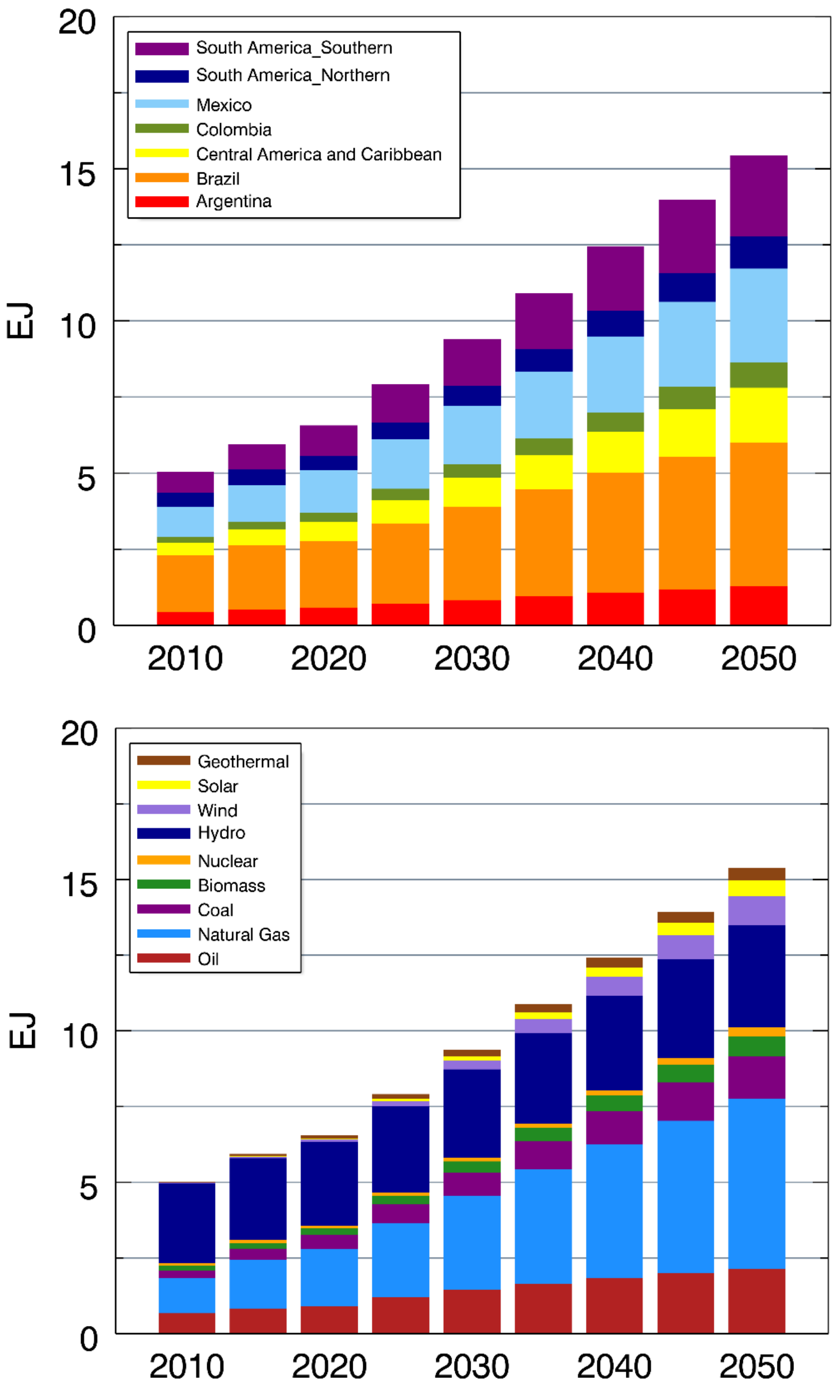

GCAM outputs for the Reference (no policy) scenario: electricity generation (EJ) by region (top) and by source (bottom).
