# Supplementary material for: The Paris pledges and the energy-water-land nexus in Latin America: Exploring implications of greenhouse gas emission reductions
Source: PLoS One. 2019 Apr 16;14(4):e0215013. doi: 10.1371/journal.pone.0215013 (PMC6467372; doi:10.1371/journal.pone.0215013)
Supplement: S1 Table — (DOCX) [file pone.0215013.s007.docx]

Regional Net GHG emissions (MtCO_2_e)^a^: Reference Scenario.

| **GCAM Region** | **2010** | **2020** | **2030** | **2040** | **2050** |
| --- | --- | --- | --- | --- | --- |
| **Argentina** | 736 | 416 | 493 | 550 | 580 |
| **Brazil** | 2181 | 1569 | 1999 | 2209 | 2050 |
| **Colombia** | 124 | 236 | 316 | 424 | 444 |
| **Mexico** | 708 | 790 | 943 | 1051 | 1153 |

^a^Global Warming Potentials (GWPs) following official NDC submissions: Brazil and Mexico

established GWPs from the IPCC Fifth Assessment Report (AR). Argentina and Colombia

defined GWPs from the Second AR.
