# Supplementary material for: The Paris pledges and the energy-water-land nexus in Latin America: Exploring implications of greenhouse gas emission reductions
Source: PLoS One. 2019 Apr 16;14(4):e0215013. doi: 10.1371/journal.pone.0215013 (PMC6467372; doi:10.1371/journal.pone.0215013)
Supplement: S1 Text — (DOCX) [file pone.0215013.s009.docx]

**Assumptions about Emissions Constraints in the Latin America and the Caribbean Region in the NDC Policy Scenarios**

As noted in the main text, this study explores two climate change mitigation policy scenarios: NDC_FullTech and NDC_NOCCS. Both scenarios are based on the ‘Paris-Increased Ambition’ scenario developed in [1], in which the main assumptions are: (1) countries achieve their NDCs through 2030; and (2) beyond 2030 CO_2_ emissions intensities decrease at annual rates implied by the NDCs or 5 percent per year, whichever is higher. However, for the Latin America and the Caribbean (LAC) region, the emissions constraints were revised based upon the supporting sources and assumptions listed below.

Table 1. Supporting Sources and Assumptions by GCAM Region in LAC.

| **GCAM Region** | **Long-term (post-2030) Goal** | **Notes** |
| --- | --- | --- |
| Argentina | The conditional NDC target is assumed to be achieved by 2050 | Emissions constraints revised according to the First Revision of Argentina’s NDC [2] |
| Brazil | Emissions constraints extrapolated from 2025-2030 rate of emissions reduction until 2050 | Revised emissions constraints based on [3] and [4] |
| Central America and Caribbean | 5% annual rate of improvement in GHG emissions per unit of Gross Domestic Product (GDP) beyond 2030 |  |
| Colombia | 2050 long-term target (30% reduction in all GHG below BAU) based on [5] | Revised emissions constraints based on [5] |
| Mexico | Emissions extrapolated from 2030 NDC emissions level towards the 2050 target stipulated in [6] (50% reduction of GHGs in 2050 compared to Mexico’s 2000 emissions: about 311 MtCO2_e_ in 2050) | Revised emissions constraints based on [4] and [6] |
| South America_Southern | 5% annual rate of improvement in GHG emissions per unit of Gross Domestic Product (GDP) beyond 2030 |  |
| South America_Northern | 5% annual rate of improvement in GHG emissions per unit of Gross Domestic Product (GDP) beyond 2030 |  |

**References**

1. Fawcett AA, Iyer GC, Clarke LE, Edmonds JA, Hultman NE, McJeon HC, et al. Can Paris pledges avert severe climate change? Science. 2015; 350:1168–1169. doi: 10.1126/science.aad5761.
2. Republic of Argentina. First Revision of its Nationally Determined Contribution [Internet]. 2016. Available from: http://www4.unfccc.int/ndcregistry/PublishedDocuments/Argentina%20First/Traducción%20NDC_Argentina.pdf
3. MMA. Fundamentos para a elaboração da Pretendida Contribuição Nacionalmente Determinada (NDC) do Brasil no contexto do Acordo de Paris sob a UNFCCC [Internet]. Brasilia[;](https://www.google.com/search?q=Brasilia&stick=H4sIAAAAAAAAAOPgE-LSz9U3qMirzEmLV-IAsQ0zKuO1NDLKrfST83NyUpNLMvPz9POL0hPzMqsSQZxiq_TEoqLMYqBwRiEAkLLn1EIAAAA&sa=X&ved=2ahUKEwi46KbP6I3eAhWPTt8KHQCTDXkQmxMoATASegQIBxAZ) 2016. Available from: <http://www.mma.gov.br/images/arquivos/clima/convencao/indc/Bases_elaboracao_iNDC.pdf>
4. Forsell N, Turkovska O, Gusti M, Obersteiner M, den Elzen M, Havlik P. Assessing the INDCs’ land use, land use change, and forest emission projections. Carbon Balance and Management. 2016; 11: 26. doi: 10.1186/s13021-016-0068-3.
5. Cadena A, Bocarejo JP, Rosales R, Arguello R, Delgado R, Flórez E, et al. Upstream analytical work to support development of policy options for mid- and long-term mitigation objectives in Colombia (Anexo A – Documento Técnico de soporte para la iNDC colombiana, pp. 27). [Internet]. Bogot[á](https://en.wikipedia.org/wiki/Bogot%C3%A1); 2015. Available from: http://www.minambiente.gov.co/images/cambioclimatico/pdf/documentos_tecnicos_soporte/Contribución_Nacionalmente_Determinada_de_Colombia.pdf
6. INECC-SEMARNAT. First Biennial Update Report to the United Nations Framework Convention on Climate Change. [Internet]. Mexico City; 2015. Available from: http://unfccc.int/files/national_reports/non-annex_i_parties/ica/technical_support_for_the_ica_process/application/pdf/executive_summary.pdf
