# Supplementary material for: The Paris pledges and the energy-water-land nexus in Latin America: Exploring implications of greenhouse gas emission reductions
Source: PLoS One. 2019 Apr 16;14(4):e0215013. doi: 10.1371/journal.pone.0215013 (PMC6467372; doi:10.1371/journal.pone.0215013)
Supplement: S2 Text — (DOCX) [file pone.0215013.s010.docx]

**Main Results from the Ancillary NDC Policy Scenarios: Long-Term Limited Nuclear Energy Capacity in Latin America**

As noted in the main text, this study explores two climate change mitigation policy scenarios: NDC_FullTech and NDC_NOCCS. Moreover, we have performed supplementary policy runs in order to test the sensitivity of our results to a long-term limited capacity of expansion of nuclear energy in Latin America. The motivation for presenting this additional analysis is the relatively high long-term deployment of nuclear energy in Brazil and, particularly, in Mexico under the NDC_NOCCS scenario, which may be argued as a high deployment pathway compared with the current role of nuclear energy in LAC (discussed in the main text).

In the supplementary simulations, the share of nuclear energy in the electricity mix are kept fixed at 2015 levels in the four focus countries of this study. This means that GCAM was run under two additional scenarios identical to the two original NDC simulations, except for the fact that nuclear energy was not allowed to expand over the following decades remaining fixed at 2015 levels in LAC. These two additional scenarios are labeled: “NDC FullTech_NOnewNUC” and “NDC NOCCS_NOnewNUC”, and are related to the two original NDC runs as follows.

Table 1. Overview of all NDC Policy Scenarios

| **Policy Scenarios by Technology Availability** | | |
| --- | --- | --- |
|  | **CCS available** | **CCS unavailable** |
| New nuclear plants are deployed | NDC FullTech* | NDC NOCCS* |
| No new nuclear plants are deployed | NDC FullTech_NOnewNUC** | NDC NOCCS_NOnewNUC** |

* Original policy scenarios explored in the main text

** Supplementary policy scenarios

A comparison of the results from the two ancillary runs against the original NDC runs showed that limiting future nuclear energy expansion in LAC did not affect the broad nature of the key nexus trade-offs and synergies discussed in the main text. In the energy domain, the absence of nuclear energy was compensated by other energy sources, notably CCS and the renewables solar and wind (a more marked expansion of solar and wind occurred in the NDC NOCCS_NOnewNUC due to the absence of CCS). A more relevant implication of the ancillary experiments could be seen in the water sector under the NDC NOCCS_NOnewNUC scenario. In this case, the water savings in the power sector relative to the reference case were larger than in the NDC_NOCCS scenario since the adverse effect of the water-intensive nuclear energy is largely minimized. In the NDC FullTech_NOnewNUC, no major change in water withdrawals differences could be noted because the water savings induced by the larger share of wind and solar in the electricity mix were offset by the increase of CCS. In the Fig 1 below, we summarize the key results from the water sector (compare this figure with Fig 4 in the main text).

| 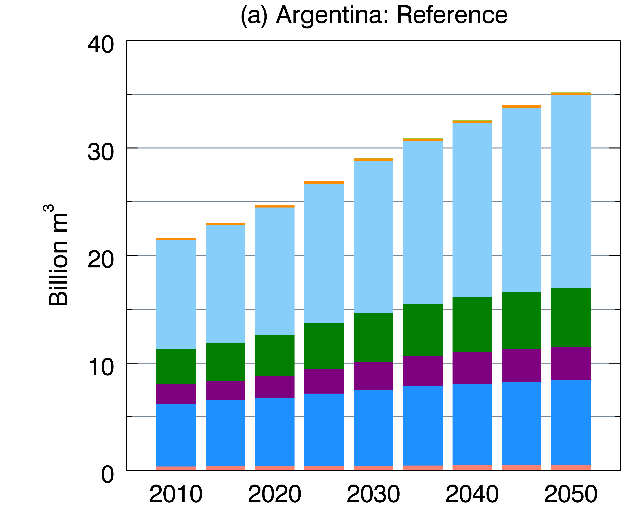 | 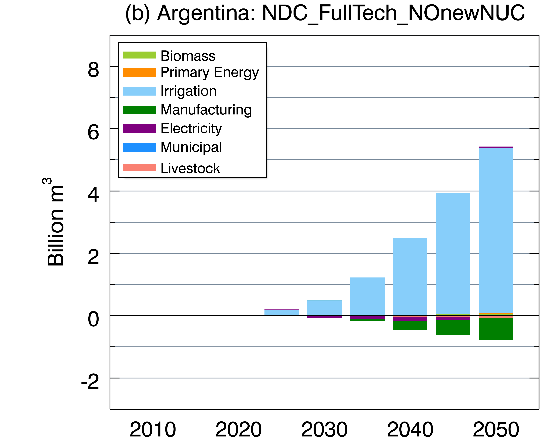 | 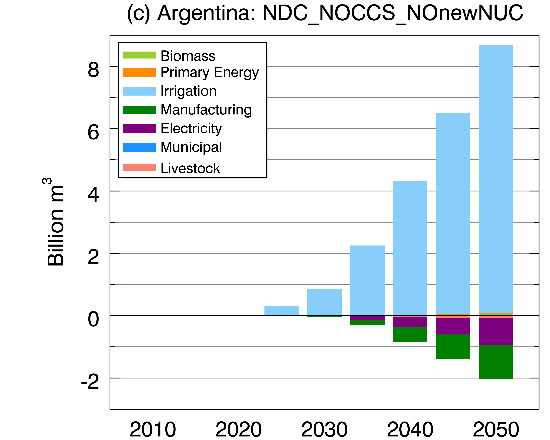 |
| --- | --- | --- |
| 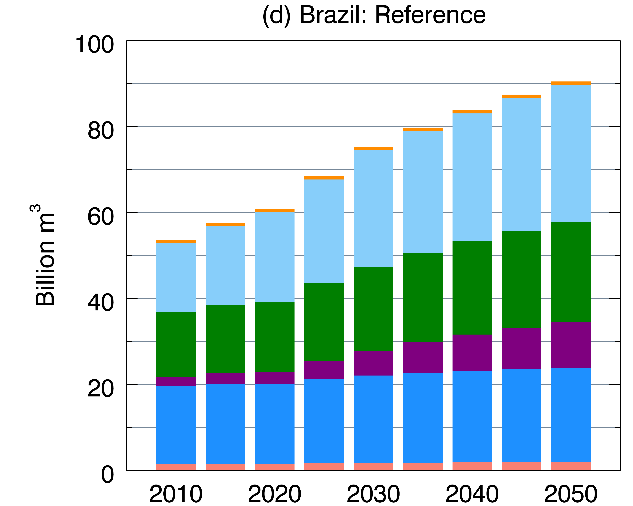 | 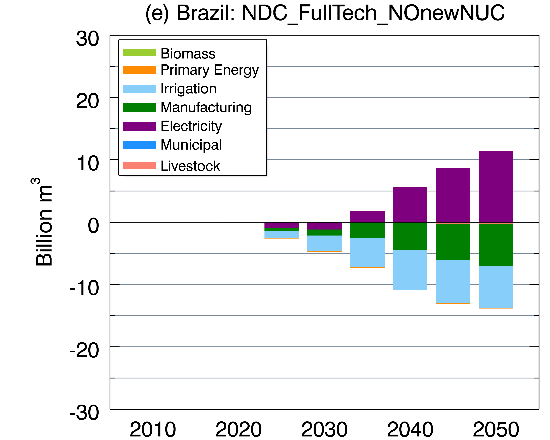 | 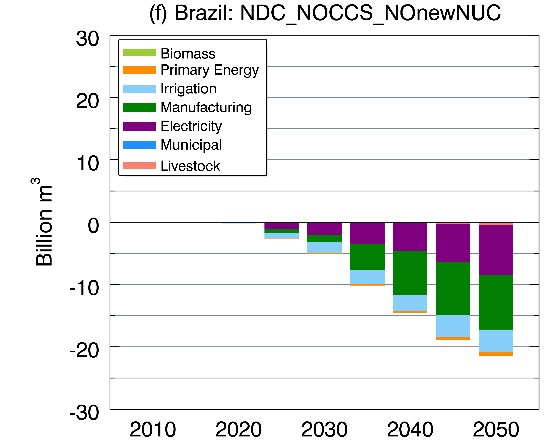 |
| 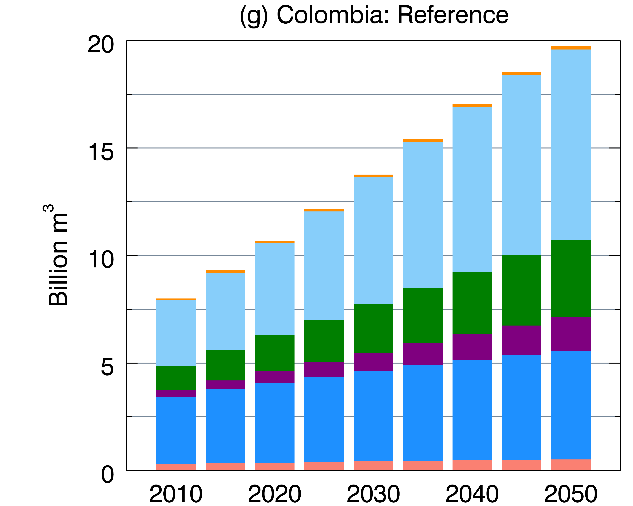 | 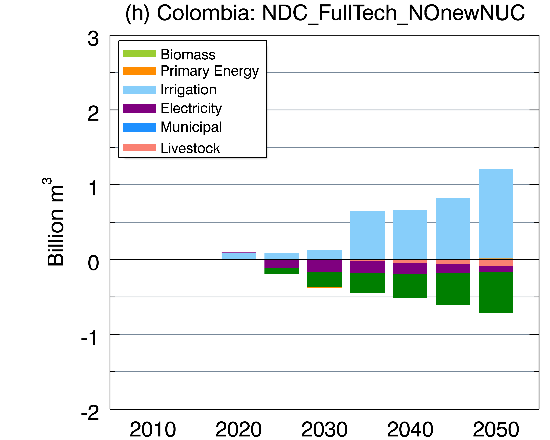 | 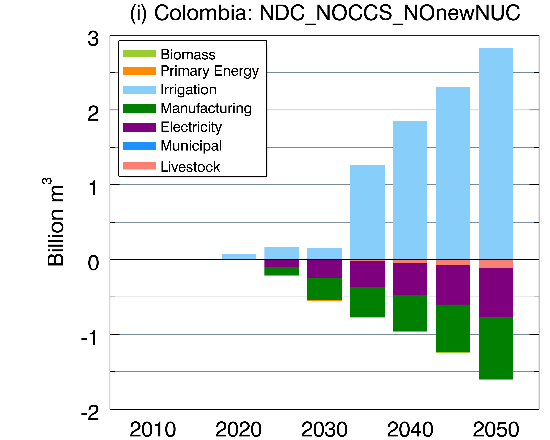 |
| 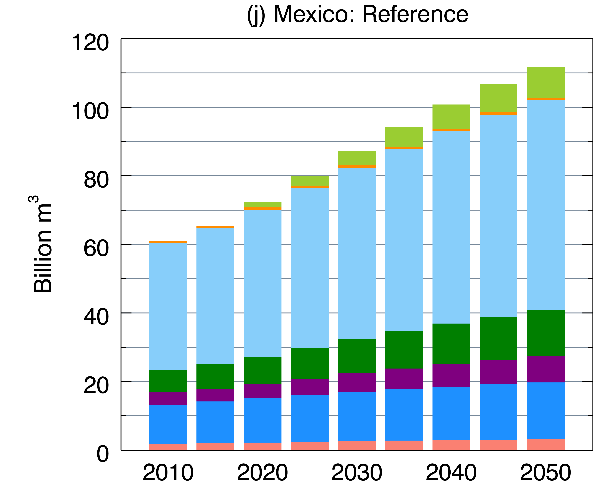 | 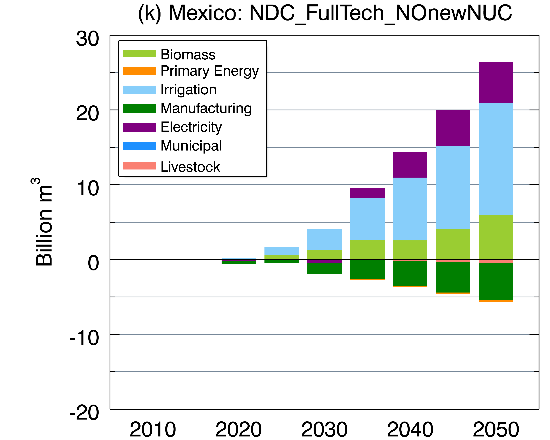 | 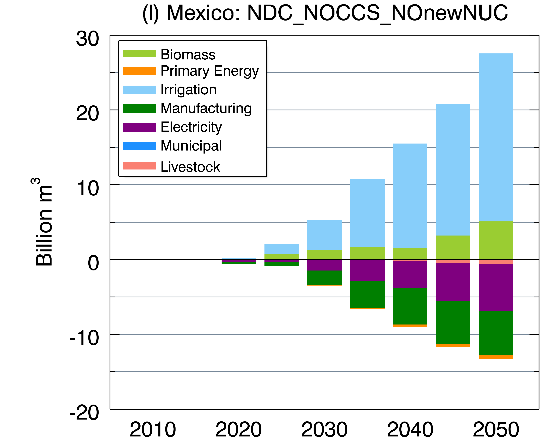 |
| Fig 1. Total water withdrawals by sector (billion m^3^) under the Reference ((a), (d), (g), and (j)). Water withdrawal differences between the NDC_FullTech_NonewNUC and the reference pathways in (b) Argentina, (e) Brazil, (h) Colombia, and (k) Mexico. Water withdrawal differences between the NDC_NOCCS_NonewNUC and the reference pathways in (c) Argentina, (f) Brazil, (i) Colombia, and (l) Mexico. | | |
